# Scaling of agent-based models to evaluate transmission risks of infectious diseases

**DOI:** 10.1038/s41598-022-26552-w

**Published:** 2023-01-02

**Authors:** Peter J. Thomas, Aidan Marvell

**Affiliations:** School of Engineering, University of Warwick, Gibbet Hill Road, Coventry, West Midlands CV4 7AL UK

**Keywords:** Diseases, Infectious diseases

## Abstract

The scaling behaviour of agent-based computational models, to evaluate transmission risks of infectious diseases, is addressed. To this end we use an existing computational code, made available in the public domain by its author, to analyse the system dynamics from a general perspective. The goal being to obtain deeper insight into the system behaviour than can be obtained from considering raw data alone. The data analysis collapses the output data for infection numbers and leads to closed-form expressions for the results. It is found that two parameters are sufficient to summarize the system development and the scaling of the data. One of the parameters characterizes the overall system dynamics. It represents a scaling factor for time when expressed in iteration steps of the computational code. The other parameter identifies the instant when the system adopts its maximum infection rate. The data analysis methodology presented constitutes a means for a quantitative intercomparison of predictions for infection numbers, and infection dynamics, for data produced by different models and can enable a quantitative comparison to real-world data.

## Introduction

Agent-based models are tools to describe, and computationally simulate, actions and interactions of autonomous individuals. The models predict global outcomes for quantities of interest, resulting from repeated interactions of agents within a population. The overall objective being to search for explanatory insight into the complex dynamics underlying the agent interactions.

Such models are often used in epidemiology to simulate the spread of infectious diseases among a population (cf.^1–15^). Data obtained with agent-based computational codes are frequently documented in the form of raw data, such as displaying the number of infected agents as a function of the computational iteration step. Examples for this can be found in Refs.^1–15^ and numerous other publications.

However, considering the number of infected agents as a function of the iteration step does not enable evaluating the scalability of the results beyond the particular cases that were simulated. Nevertheless, this issue is essential in the context of establishing in how far the model can yield explanatory insight and as regards a quantitative data comparison with results of other models or field data.

A recent article^1^ introduced a new agent-based model to evaluate the transmission risks of infectious diseases in facilities and the author has kindly made his computational code available^16^. That provided the opportunity to use it, with the aim to investigate the fundamental, underlying system dynamics and assess the scalability of the model in terms of a generalized analysis.

The approach adopted here leads to closed-form expressions summarizing the system development. It reveals that the dynamics for the temporal growth of the infected agents can be described by two parameters. One of these is a scaling parameter characterizing the global population dynamics of the system. The other one identifies the instant of the maximum infection rate. This second parameter becomes relevant for small populations where interaction probabilities are low.

Knowledge of how these two parameters vary with the population size is sufficient to characterize the entire system development. It enables a direct quantitative intercomparison between data from different models, such as those described in Refs.^1–15^, as well as numerous other publications and field data.

## The model and the boundary conditions

### The original model

The model underlying the results discussed here is described in detail in Ref.^1^. Therefore, its nature is only briefly outlined, and the reader is referred to the original publication for details.

The model of Ref.^1^ is an agent-based model simulating interacting autonomous persons. The computational code of Ref.^1^ constitutes commands prescribing the movement of people within a closed, two-dimensional domain that is intended to represent a facility, such as a large, shared office space.

The coordinates of the positions of the agents within the computational domain are described in terms of a pair of real numbers $(x_i(n), y_i(n))$, where the index *i* identifies the agent and *n* is the iteration step of the computational code. In Ref.^1^ the dimensions of the facility in the horizontal and vertical directions are set to 300 units. That dimension, here referred to as *D*, of the computational domain was left unchanged for the current study. Using a domain of the particular size $300 \times 300$ does not affect the generality of the results obtained. That is so, because all position data $(x_i(n), y_i(n))$, and the contagion radius, *h*, are real numbers. They could, therefore, be normalized as $(x_i(n)/D, y_i(n)/D)$, and *h*/*D*, to map them onto intervals [0, 1]. Thus, the results must be independent of the domain size $D \times D$. Since no direct reference to the position data will be made throughout the current discussion the exact values of $(x_i(n), y_i(n))$ are not relevant here.

At the beginning of a simulation the computational domain is initialized by populating it with susceptible agents of prescribed population size *P*, positioned at random locations. In all cases simulations are initiated with one single infected person within the computational domain. That agent will be referred to as the infection seed herein. The particular position of the infection seed is also determined randomly in the original code. However, for some of the results presented here the initial position of the infection seed was prescribed. The rationale for this will be addressed in connection with the discussion of the effects of different boundary conditions on the results obtained.

The code specifies rules that determine, at each computational step, if an agent moves or remains in its place. If it moves it is probabilistically determined how far, and in which direction, the motion occurs. The model distinguishes between two different movement types, these are local and long-distance displacement. The movement type to be performed by each agent, at each computational step, is also determined probabilistically. It is assumed that local displacements are more frequent than long-distance movements. Here only simulations with local displacements are considered because the primary purpose is to demonstrate the methodology by which the simulation data are analysed to obtain results of general nature.

The code of Ref.^1^ defines a probabilistic distribution that determines whether a susceptible person becomes infected when it enters a certain specified neighbourhood of a contagious person. The probability of infection can be different for each person to represent a heterogeneous population. The number of infected *I*, and susceptible agents *H*, is monitored as a function of the computational iteration step, *n*, with $P = I(n) + H(n)$. For the current discussion the particular iteration step at which the constant state $I(n) = P$ is reached is referred to as the saturation step, $N_S$, in the remainder of this article. Unless otherwise specified the population size, when referred to in the text, includes the infection seed.

A small issue of the computational code for Ref.^1^, available at^16^, was noticed and modified. It was found that once a healthy agent had entered the contagion region of an infected agent it became fixed in that location until the infected agent had moved away from the healthy one. This was due to there being no movement commands in the *’else’* block in lines 127-137 of the code at Ref.^16^. That was felt to be unrealistic. Because an agent that is unaware of being within the contagion region of an infected agent would not remain there until the infected agent has repositioned itself. The agent would move to a new position whenever it desired to do so and independently of the action of the infected agent. Therefore, the code was modified such that this issue is resolved for the current simulations by enabling the agent to move, irrespective of the action of the infected agent.

### Raw data

**Figure 1:**
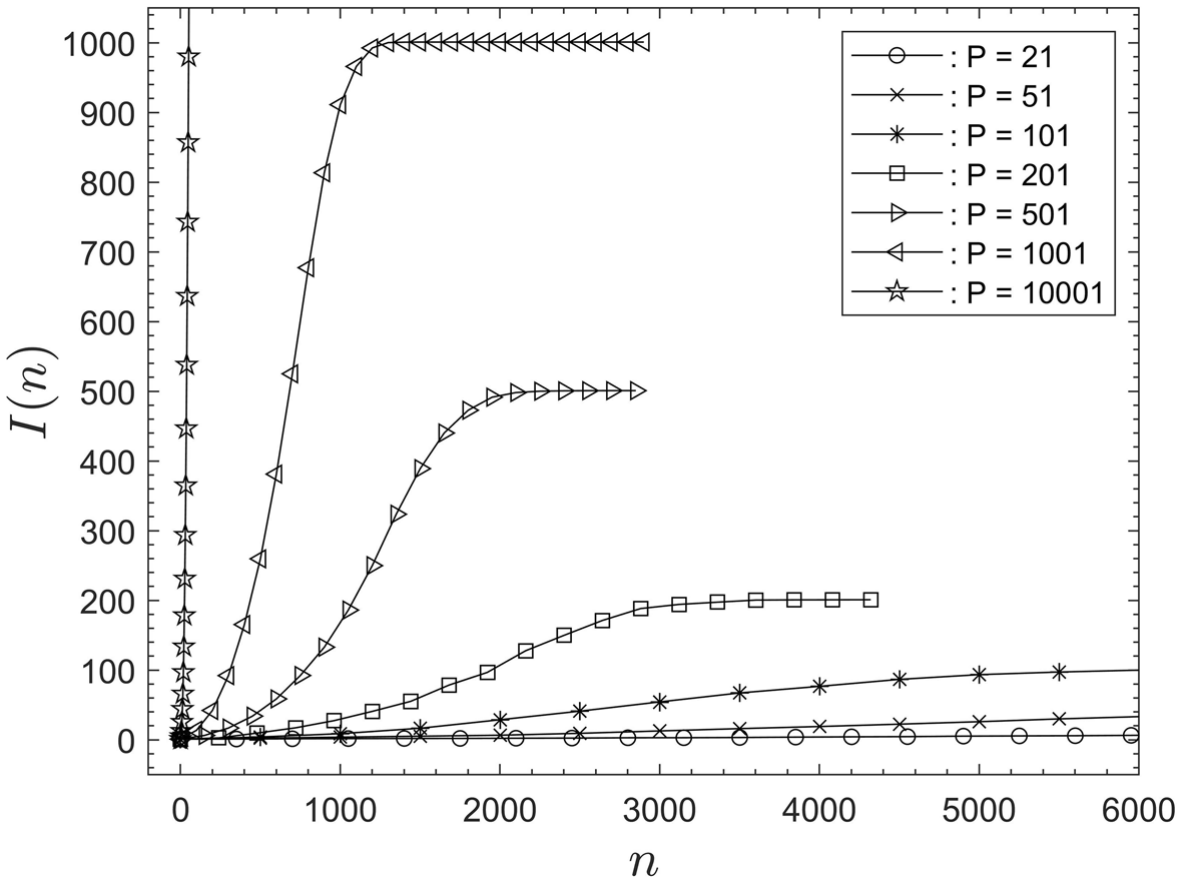
Raw data for the number of infected people, *I*(*n*) , as a function of the iteration step, *n*, for seven different, individual simulations and population sizes *P*. Only a fraction of the available data points, for each population size, are shown.

Figure 1 displays examples of raw data sets obtained with the computational code of Ref.^1^. The figure shows the number of infected people *I*(*n*) as a function of the iteration step *n*. The data are for individual simulations, for seven different population sizes between 21 and 10001. The remaining independent input parameters for the simulations in Fig. 1 are summarized in Appendix A, and they remained constant for all simulations discussed herein. For each simulated population size in Fig. 1 only a small fraction of all available data points, *I*(*n*) , are displayed for the sake of clarity (similarly for Figs. 3 and 4). The total number of data points in Fig. 1 is, for instance, $n = 25000$ for $P = 21$ and $n = 218$ for $P = 10001$.

In Ref.^1^ data sets for thirty simulations, for any particular set of constant input parameters, were used for the averaging process of data. It was found that averaging fifteen data sets are sufficient to reproduce the data of Ref.^1^ within a tolerance of one standard deviation (cf.^17^). Moreover, averaged data of fifteen repetitions of simulations are sufficient to uncover the general scaling issues to be discussed.

The format in which the data in Fig. 1 are presented is that used throughout Ref.^1^, and in many other publications, such as^2–15^. The main goal here is to find an alternative format suitable to collapse the data and find appropriate interpolations that yield closed expressions for the summary of the results. However, prior to progressing towards that main goal it is required to briefly address the boundary conditions implemented in the model of Ref.^1^.

In Ref.^1^ the boundary conditions are such that agents cannot cross the limits of the computational domain. It is argued that this models the rigid wall of a facility. If an agent gets a motion allocated that would take it across the system boundary, then the motion during that iteration step is ignored and the agent remains in its current location. The agent will remain there until a subsequent iteration step allocates a motion directed away from the wall. This original mode of boundary condition is referred to as the rigid-wall boundary condition (RWBCs) in the remainder.

### Periodic boundary conditions

The fact that the RWBCs of Ref.^1^ can result in agents remaining in fixed positions or become temporarily locked to movements within the immediate vicinity of the system boundary, due to probabilistic motion allocations, was felt to be unrealistic. A person in an office environment would not behave in that manner.

Moreover, RWBCs result in a data bias as regards the fundamental dynamics governing the system development. That is, because computations started with the infection seed initially positioned near a boundary will result in a different temporal development compared to results for an infection seed that was initially located in the centre of the computational domain.

In order to study the fundamental dynamics of the model it is therefore desirable to use boundary conditions that can be expected to produce results that are independent of the initial location of the infection seed. Therefore, the code Ref.^1^ was modified by introducing periodic boundary conditions (PBCs), in order to establish a homogeneous computational domain. Results obtained from running the code for both sets of boundary conditions will be compared to each other in “Comparison of results for RWBCs with PBCs” section, before progressing to discuss the remaining results based on simulations with PBCs only.

**Figure 2:**
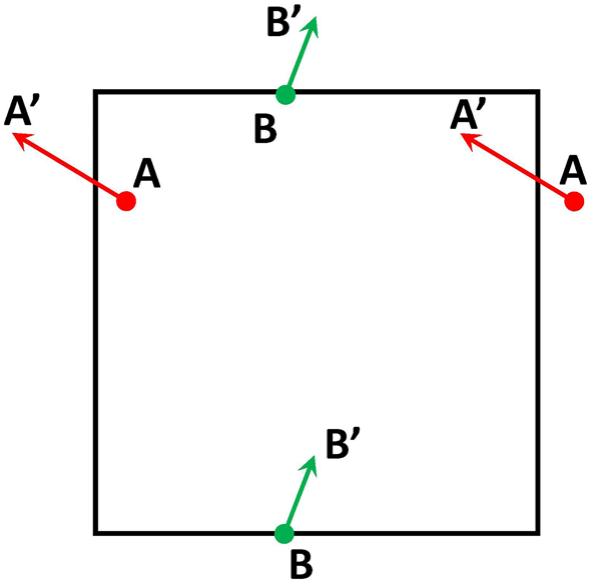
Schematic illustration of the periodic boundary conditions.

The PBCs implemented in the model are illustrated in Fig. 2. The figure displays how agents can exit the computational domain at its boundaries to immediately reappear at a corresponding position at the opposite boundary. If, for instance, the agent *A*, located near the top of the left boundary in Fig. 2, were required to move a certain distance in the direction of the arrow shown, and if this movement would take it across the system boundary, then that agent would be reintroduced immediately, as part of that iteration step, into the system at the location $A'$ at the right boundary. The corresponding process would be performed for agent *B* at the top and bottom boundaries of the domain in Fig. 2.

Similar to the RWBCs of Ref.^1^ the PBCs illustrated in Fig. (2) can also be considered to represent a facility with a rigid wall. That is, because simulations with PBCs also represent a closed system. Nevertheless, the advantage of the new PBCs is that these do not artificially modify the underlying system dynamics as a result of agents becoming locked to the wall-near region for, potentially, a number of successive iteration steps.

### Comparison of results for RWBCs with PBCs

**Figure 3:**
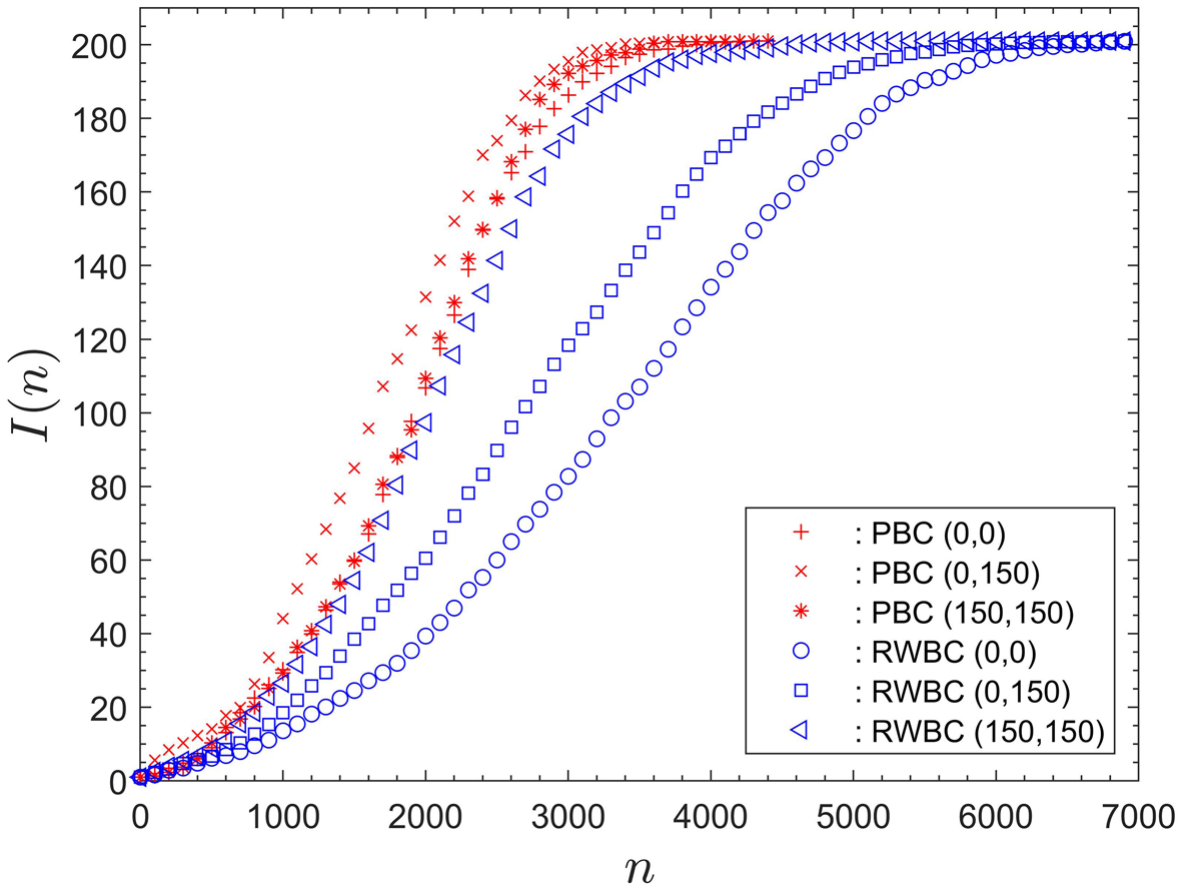
The number of infected, *I* as a function of the iteration step *n*, for different starting positions (0, 0) , (0, 150) and (150, 150) . Comparison of periodic boundary conditions to rigid-wall boundary conditions.

Figure 3 compares results obtained for RWBCs and PBCs, for a population $P = 201$ and for otherwise identical conditions, as summarized in the table in Appendix A. Figure 3 contains data for three different starting positions for the infection seed. The position (0, 0) corresponds to the lower left-hand corner of the computational domain, (0, 150) refers to the middle of the left boundary and (150, 150) to the centre of the computational domain.

The comparison of the data for PBCs and RWBCs in Fig. 3 shows that PBCs substantially reduce the number of iteration steps required to reach saturation. For RWBCs the saturation iterations step, $N_S$, is a broad interval ${\mathcal {O}}(4600-6800)$. For PBCs it is a narrow region ${\mathcal {O}}(3200-3400)$. Thus, using PBCs reduces the output variability and almost halves the number of iteration steps required to reach saturation.

Most importantly, the data comparison in Figs. 3 reveals that PBCs homogenise the computational domain. For the case of RWBCs the outcome of the simulations depends strongly on the starting location of the infection seed. For instance, for RWBCs the difference in the number of infected at $n \approx 3000$, for starting location (0, 0) and (150, 150) , is ${\mathcal {O}}(100)$. That corresponds to $50 \%$ of the entire population. For PBCs at a corresponding location, approximately halfway through the system development to saturation, at $n \approx 2000$, the difference between the corresponding curves is only ${\mathcal {O}}(20)$. In fact, the curves for starting location (0, 0) and (150, 150) for PBCs in Fig. 3 are essentially identical.

Thus, the main conclusion is that PBCs homogenise the computational domain. They are, therefore, preferable in the context of analysing the fundamental system dynamics and they will be used throughout the remainder.

## Data rescaling

### Selection of interpolation function

The data representation in Fig. 1 only permits qualitative comments. Due to the different population sizes the data appear very different, apart from sharing a similar, overall shape. The goal is to find an alternative format that enables drawing conclusions of a general nature. To this end it is initially required to rescale the data for *n* and *I*(*n*) .

Figure 1 shows that the data sets display typical sigmoidal characteristics. That is, the data are represented by an S-shaped profile. Sigmoid functions are often used to model system dynamics when no specific mathematical model exists (cf.^18^). A curve frequently used to model sigmoidal data distributions is the hyperbolic tangent function. The current data analysis and discussion will be based on that type of function.

### Removing redundant data

The first step involved in rescaling the data is the averaging process (cf. “The original model” section) for each set of fifteen individual simulations, containing the same number of *N* iteration steps. This yields mean data with qualitatively similar sigmoidal characteristics as displayed by the individual data sets in Fig. 1.

Reference to Fig. 1 reveals that each data set, for these individual runs, contain substantial numbers of data points for which the system is saturated, that is $I(n) = P$. These data will be referred to as redundant data points. Following the data averaging the mean curve also contains redundant data points. These are removed by truncating the averaged data sets at their associated saturation step, $N_S$. Thus, all data, of the averaged curves with $n > N_S$, are deleted. As a consequence, each averaged, truncated data set will have a different data length $N_S$. Note also that Fig. 1 shows that the saturation step $N_S$ occurs earlier for larger populations.

### Remapping iteration steps

The iterations $n = 0 \ ... \ N_S$, of the truncated data sets, are mapped onto the interval $[-1, 1]$. This is achieved by introducing a rescaled iteration number ${\tilde{n}}$ as

1 $$\begin{aligned} {\tilde{n}} = \frac{2n-N_S}{N_S} . \end{aligned}$$

That expression yields ${\tilde{n}} = -1$ for $n = 0$, and ${\tilde{n}} = 1$ for $n = N_S$.

The particular choice of mapping in Eq. (1) means that, in an ideal system, that exactly follows a hyperbolic tangent scaling, the origin with ${\tilde{n}} = 0$, corresponds to the instant where the highest infection rate is adopted.

Similar to *n* in Eq. (1), the number *I*(*n*) of infected people is rescaled as

2 $$\begin{aligned} I^*(n) = \frac{2I(n)-P}{P} . \end{aligned}$$

Equation (2) maps $I = 0 \ ... \ P$ onto the interval $[-1,1]$, with $I^*(n) = -1$ for $I(n) = 0$ and $I^*(n) = 1$ for $I(n) = P$. Note that, for computing numeric values for $I^*(n)$, the infection seed will be excluded from *I*(*n*) and *P* in Eq. (2).

### Closed-form data description based on hyperbolic-tangent function

The hyperbolic-tangent data interpolations, suggested in "Selection of interpolation function" section, yield closed-form expressions suitable to describe all results. These expressions will now be obtained.

In a general Cartesian coordinate system, the hyperbolic-tangent function is $y = \tanh (x)$ with $y = 0$ at $x = 0$. Scaling factors $\alpha$ and $\beta$ can be introduced as $\tanh (\alpha (x+\beta ))$. The parameter $\alpha$ adapts the data profile to the underlying characteristics of the system evolution for any particular population size *P*. The value of $\beta$ describes translations of the curve along the abscissa. That is, it accounts for simulation data that conform to a hyperbolic tangent profile, but which are shifted, such that at $x = 0$ one has $y \ne 0$. Therefore, the simulation data are summarized in the form

3 $$\begin{aligned} I^*(n) \equiv I^*({\tilde{n}}) = \tanh (\alpha ({\tilde{n}} + \beta )) \ \ . \end{aligned}$$

For simplicity the notation $\sigma = \alpha ({\tilde{n}} + \beta )$ and $\gamma = \alpha /N_S$ is introduced.

Using Eqs. (2) and (3) the infection rate is given by is given by

4 $$\begin{aligned} \frac{dI(n)}{dn} = \frac{\gamma }{\Big (\cosh (\sigma ) \Big )^2} \ \ . \end{aligned}$$

The derivative of Eq. (4) has its maximum at $\sigma = 0$. Substituting Eq. (1) into that expression for $\sigma$ and then solving for *n* yields that the maximum infection rate occurs at $n = N_{Imax} = (N_S/2) (1-\beta )$. For the case when $\beta = 0$ this gives $N_{Imax} = N_S/2$. That is, the instance when the maximum infection rate is adopted lies halfway between the iteration $n = 0$, when only the infection seed is present, and the saturation time step $N_S$. Introducing the expression for $N_{Imax}$ into Eq. (2) yields ${\tilde{n}}(N_{Imax}) = - \beta$. Thus, the value of $\beta$ represents the location of the maximum infection rate when a curve for the number of infected agents, $I^*({\tilde{n}})$, is not centered symmetrically around ${\tilde{n}} = 0$.

### The cost function

In Ref.^1^ a cost function is defined that yields a knee point $\Lambda$. It is discussed there that this represents the right decision point at which the relative value of a variable is no longer significant in terms of its final contribution. That is, the iteration step $\Lambda$ corresponds to the outbreak end point. In terms of the current nomenclature that cost function is expressed as

5 $$\begin{aligned} J(n) = \frac{I(n)}{P} -\frac{n}{N_S} \ \ . \end{aligned}$$

The cost function, in Eq. (5), possesses only one global maximum^1^. The iteration number at which this maximum occurs is the knee point. Since closed-form expressions for *I*(*n*) are known through $I^*({\tilde{n}})$, and with Eqs. (2) and (3), it is also possible to obtain a closed-form expression for the location of the knee point.

The knee-point location is found in "Knee point" section based on Eq.(5). However, for the purpose of illustrating the results, in comparison to simulation data in “Results for cost function for different population sizes” section, the cost function is here defined in terms of the rescaled variables $I^*({\tilde{n}})$ and ${\tilde{n}}$ as

6 $$\begin{aligned} J^*({\tilde{n}}) = I^*({\tilde{n}}) - {\tilde{n}} \ \ . \end{aligned}$$

### Knee point

From Eq. (5), the condition for the knee point is

7 $$\begin{aligned} \frac{dJ(n)}{dn} = \frac{1}{P} \frac{dI(n)}{dn} - \frac{1}{N_S} = 0 \ \ . \end{aligned}$$

Substituting in Eq. (4) and the expression for $\gamma$ into Eq. (7) yields after rearranging

8 $$\begin{aligned} \sqrt{\alpha } = \cosh (\sigma ) = \cosh (\alpha \left( {\tilde{n}} + \beta \right) ) \ \ . \end{aligned}$$

Solving Eq. (8) for ${\tilde{n}}$ yields the value ${\tilde{\Lambda }}$ for the knee point location in terms of rescaled iterations steps. One finds that

9 $$\begin{aligned} {\tilde{\Lambda }} = \frac{1}{\alpha } {{\,\textrm{arcCosh}\,}}(\sqrt{\alpha }) - \beta \ \ , \end{aligned}$$

and with ${\tilde{\Lambda }} = (\Lambda - N_S)/N_S$ this can be rearranged to give

10 $$\begin{aligned} \Lambda = \frac{N_S}{2} \Bigg \{ 1 + \bigg [ \frac{1}{\alpha } {{\,\textrm{arcCosh}\,}}(\sqrt{\alpha }) - \beta \bigg ] \Bigg \} \ \ . \end{aligned}$$

Equation (10) represents the knee point location in terms of the original unscaled iteration step associated with the raw data, and for any particular population for which saturation occurs at a saturation step $N_S$.

A special case of the expression in Eq. (10) arises for $\alpha = 1$ and $\beta = 0$. Reference to Eq. (10) reveals that the term in the square brackets then vanishes, since ${{\,\textrm{arcCosh}\,}}(1) = 0$. Therefore, one has $\Lambda = N_S/2$. That value for $\Lambda$ is the same as that obtained, from Eq.(4), for the instant $N_{Imax}$ when the system displays the maximum infection rate. For $\alpha = 1$ and $\beta \ne 0$ the term in the curly brackets is $1 - \beta$. This simply reflects the shift of the data when they are not distributed symmetrically around the origin. Thus, in both these cases the knee point and the instant of maximum infection rate coincide. However, for $\alpha \ne 1$ the instants of maximum infection rate and knee point do not coincide. The data from the simulations discussed below will show that $\alpha \ne 1$.

### Ethical approval

The study did not involve human participants. The authors confirm that they have adhered to all issues identified on the webpage of the *Nature - Scientific Reports.*

## Simulation results

### Results for infection behaviour for different population sizes

**Figure 4:**
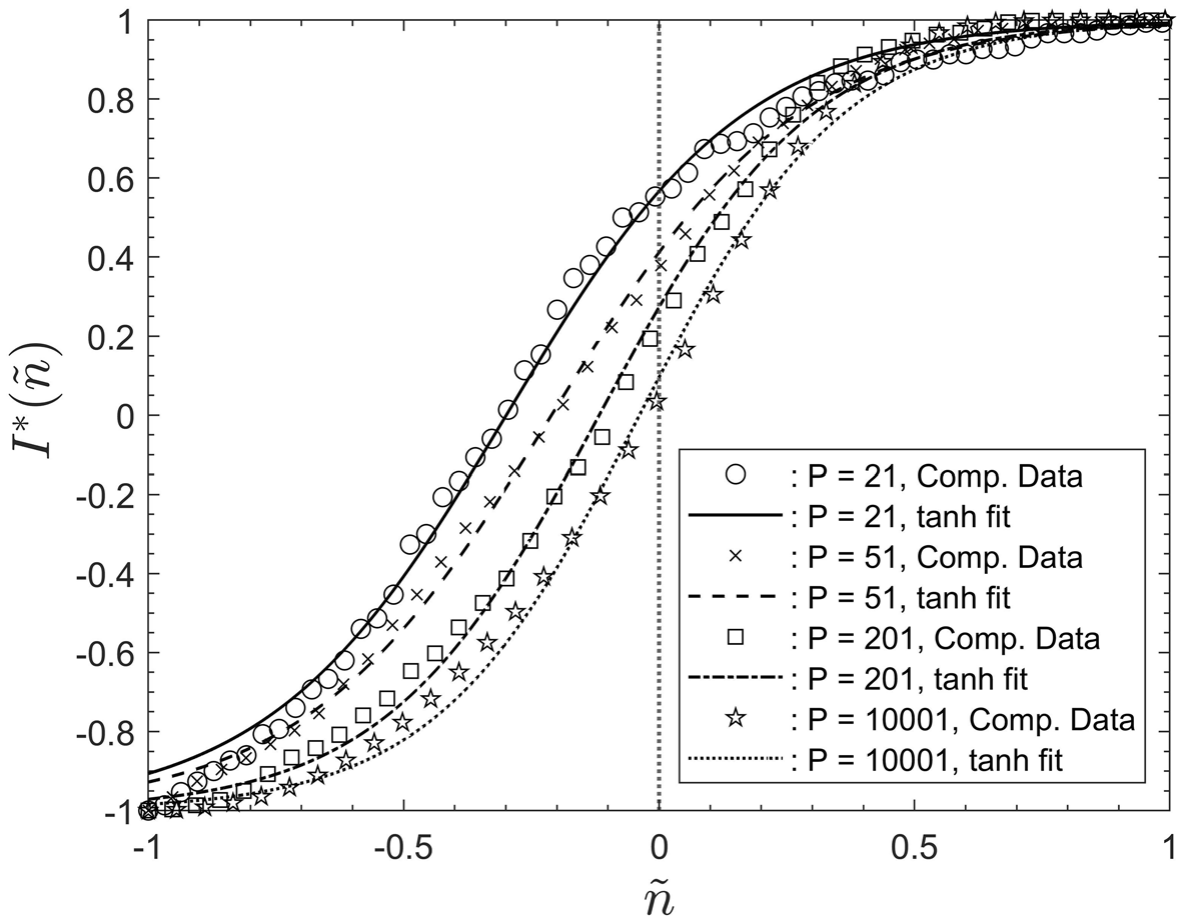
Simulation data, for $P = 21,51,201,10001$, for the number of infected people, $I^*$ as a function of the rescaled iteration step ${\tilde{n}}$, together with their associated hyperbolic-tangent data interpolations superposed.

Figure 4 displays averaged data sets for the number of infected agents, $I^*$, as a function of the rescaled iterations step ${\tilde{n}}$. Data for the population sizes $P = 21, 51, 201, 10001$ of Fig. 1 are shown. Each data set is shown in comparison to its associated hyperbolic-tangent data interpolations according to Eq. (3). The data for $P = 101, 501, 1001$ were omitted from the figure for the sake of clarity. The figure reveals that all displayed simulation data are very closely approximated by hyperbolic-tangent functions. The same is the case for the data sets for $P = 101, 501, 1001$.

Table 1 summarizes the fitting parameters $\alpha , \beta$, together with the saturation step $N_S$ and the knee-point location ${\tilde{\Lambda }}$. Reference to the data in the table reveals that the value of parameter $\alpha$ very gradually increases with the population size *P*. The value of $\beta$ decreases with *P*, approaching zero for large *P*. The number of saturation steps, $N_S$, decreases with increasing population. The reasons for this will be addressed below.

**Table 1:** Parameters of least-squares fitting, according to Eq. (3), for the data of all simulated population sizes. Also included in table is the saturation step $N_S$, and the knee point location ${\tilde{\Lambda }}$, for each population size.

P	21	51	101	201	501	1001	10001
$\alpha$	2.149	2.08	2.164	2.395	2.469	2.383	2.512
$\beta$	0.299	0.2105	0.2134	0.1171	0.1137	0.08028	0.03827
$N_S$	24987	12557	7390	4278	2976	1467	218
${\tilde{\Lambda }}$	0.134	0.226	0.219	0.302	0.301	0.34	0.374

The particular numeric values of $\alpha$ in Table 1 have no direct physical relevance associated with them. These values result from remapping the iteration steps onto the interval $[-1, 1]$. Different numeric values would have been obtained if one had mapped onto, for instance, the interval $[-\pi , \pi ]$. Nevertheless, what is of importance is that $\alpha$ increases with *P*, while $\beta$ and $N_S$ decrease.

Figure 4 shows that the data are not distributed symmetrically around ${\tilde{n}} = 0$. This is a consequence of the non-zero values of $\beta$. It was discussed in “Closed-form data description based on hyperbolic-tangent function” section  that ${\tilde{n}} = - \beta$ is the instant of the highest infection rate. Since $\beta$ approaches zero for large populations, the number of infected, $I^*$, becomes symmetrically distributed around ${\tilde{n}} = 0$.

**Figure 5:**
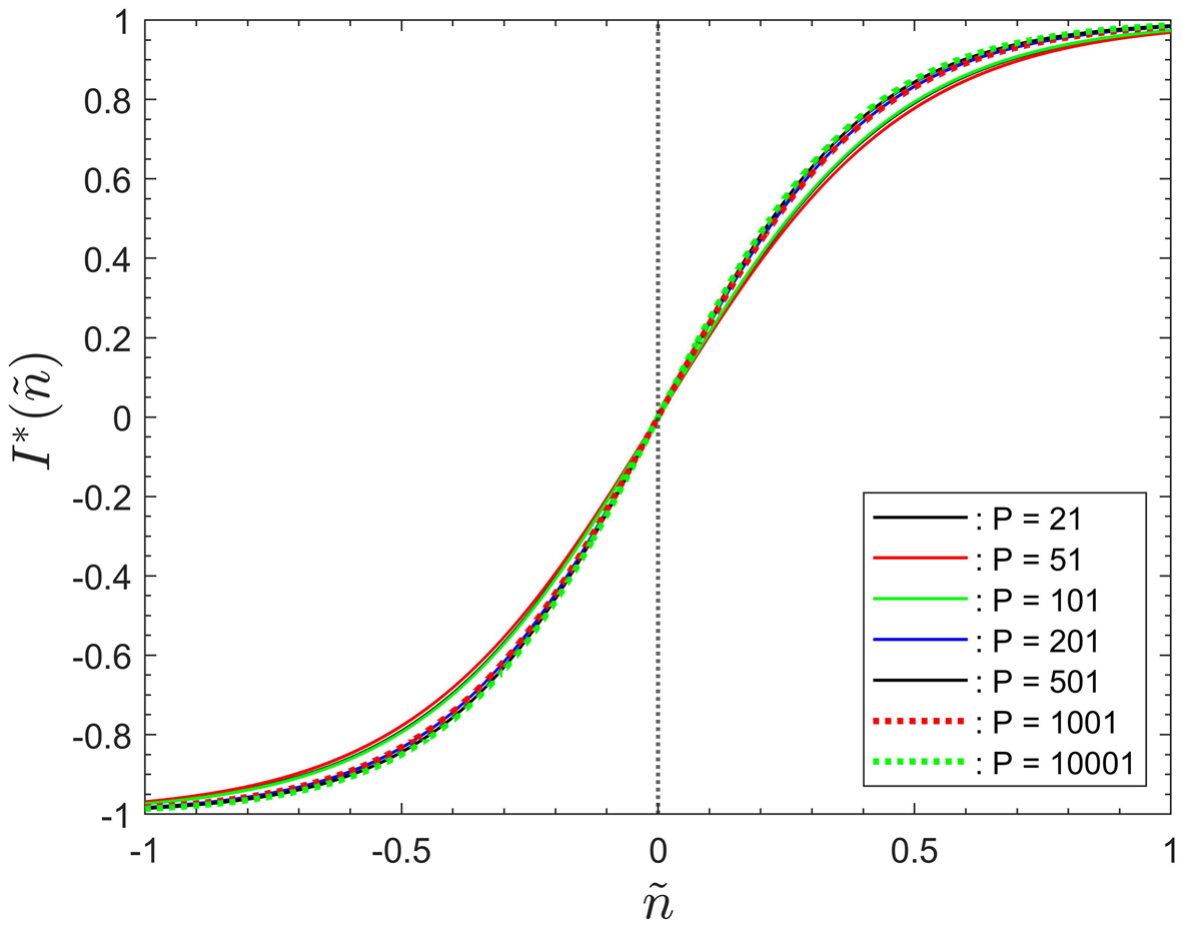
The data fits for the rescaled number of infected people, $I^*$ as a function of the rescaled iteration step ${\tilde{n}}$. Data for all simulated population sizes, after equating fitting parameter $\beta$ identically zero.

To illustrate the overall effects of parameter $\alpha$ on the shape of the curves Fig. 5 shows the data fits for all seven simulated values of *P* after equating $\beta$ identically zero. That figure now displays all data centred around ${\tilde{n}} = 0$ and all data nearly collapse onto one another. However, the data collapse is not complete. This reflects the very gradual increase of $\alpha$ with the population size. Note that the overall shape of the curves would approach the shape of a step function, indicated by the dotted line in Fig. 5, if the fitting parameter $\alpha$ were to approach infinity.

The overall behaviour displayed by Fig. 5 implies that $\alpha$ constitutes the characteristic scaling parameter for the entire system. This is one of the main results of the current study. The parameter $\alpha$ represents a scaling factor for time, when represented in terms of the iteration number ${\tilde{n}}$.

It would probably be possible to use other sigmoidal functions, incorporating more than two fitting parameters, to further improve the quality of the data fits in Fig. 4. However, the main result is that rescaling the data as above, and then using hyperbolic-tangent least squares fits, with only two scaling parameters, is sufficient to provide a very good representation, together with closed-form expressions, for the output data of the type of model discussed here.

### Results for cost function for different population sizes

**Figure 6:**
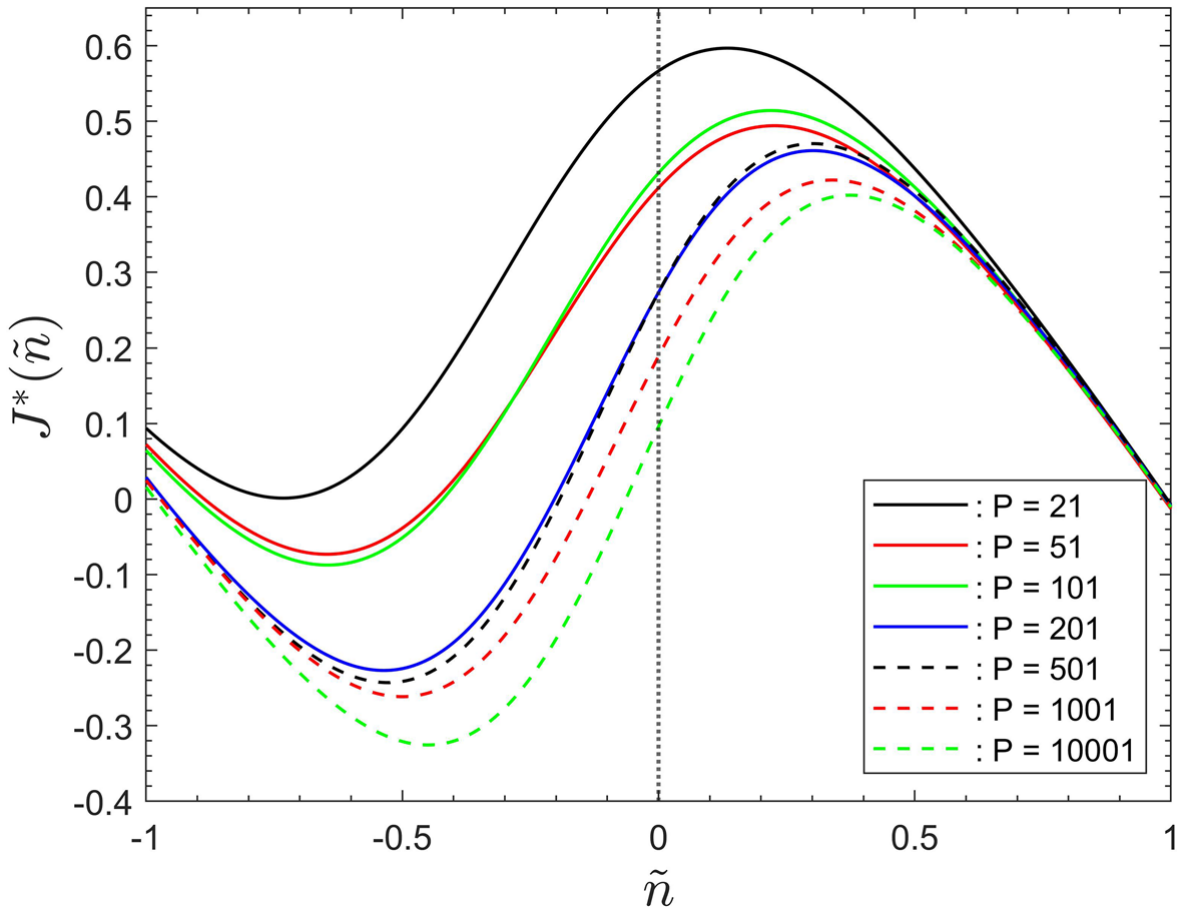
The cost function, $J^*({\tilde{n}})$, for different population sizes, as a function of the rescaled iterations step ${\tilde{n}}$.

**Figure 7:**
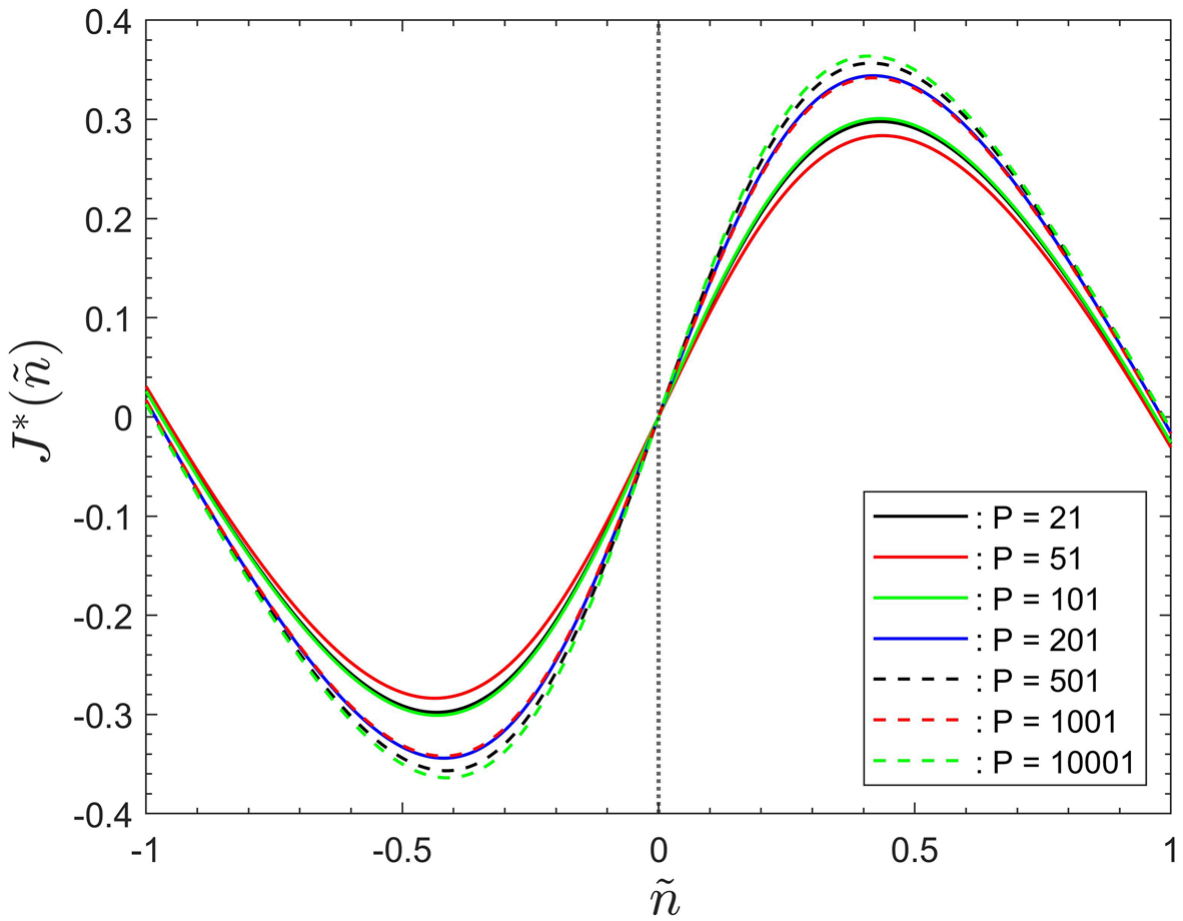
The cost function, $J^*({\tilde{n}})$, for different population sizes, as a function of the rescaled iterations step ${\tilde{n}}$, after equating $\beta$ identically zero.

Figures 6 and 7 display the cost function of Eq. (6) associated with the data for the number of infected agents for the seven population sizes simulated.

Corresponding to Fig. 4 the data in Fig. 6 show the results based on the data fitting of Eq. (3), with both fitting parameters, $\alpha$ and $\beta$, included. For the purpose of illustrating the effects of parameter $\alpha$ Fig. 7 redisplays the data of Fig. 6 after equating parameter $\beta$ identically zero. But note that neglecting $\beta$ here effects the shape of the cost function, in comparison to their corresponding curves in Fig. 6. That is, because the term $I^*({\tilde{n}})$, on the right hand side of Eq. (6), becomes shifted relative to the term ${\tilde{n}}$.

Nevertheless, it can be seen that the behaviour of the data in Figs. 6 and 7 mirrors that discussed in “Results for infection behaviour for different population sizes” section for the number of infected agents. Figure 7 shows that the global behaviour of the simulation data only depends on the fitting parameter $\alpha$. This reconfirms that it constitutes the main scaling parameter required to characterize the global system behaviour.

### Scaling results to real-world time

The data as presented in Fig. 1 do not enable relating the computational iteration step to real-world time. However, if the total length, *T*, of an outbreak were known in advance, say in terms of weeks, months or years, then Eq. (1) could be modified as

11 $$\begin{aligned} {\tilde{n}}_T = \frac{T}{2}{\tilde{n}} . \end{aligned}$$

to map the arbitrary iteration step ${\tilde{n}}$ onto the real-world time domain. Here ${\tilde{n}}_T = - T/2$ would correspond to the time for the outbreak of the disease and ${\tilde{n}}_T = T/2$ to the saturation time, when all agents are infected.

Nevertheless, in practice *T* is, of course, not known in advance. However, from “Closed-form data description based on hyperbolic-tangent function”, “The cost function” and “Knee point” sections it is known how the instant of the occurrence of the maximum infection rate $N_{Imax}$ is related to the knee point $\Lambda$. Since $\alpha > 1$ the instant $N_{Imax}$ is, necessarily, prior to $\Lambda$. Thus, it is possible, in principle, to monitor the infection rate of an outbreak and, once its maximum has been observed, use Eq. (10) to find $\Lambda$. However, this would necessitate evaluating the parameters $\alpha$ and $\beta$ in Eq. (3) from an incomplete data set, ending at the instant of $N_{Imax}$. This would, evidently, introduce uncertainties into any predictions.

The value of $\alpha$ could, in principle, also be determined from a data fit to incomplete field data sets with $n < N_{Imax}$, or $n > N_{Imax}$. However, for data with $n < N_{Imax}$ the data fit would become increasingly unreliable for decreasing data-set length. For $n > N_{Imax}$ the data fit improves. However, the closer one has approached the saturation level the less practical need there will exist for a prediction regarding the development during the remainder of the outbreak.

The same approach of scaling time as expressed by Eq. (11) can also be adopted when comparing results from different models, for which data are given as a function of a days, months or years, when it is desired to intercompare computations for time periods of different lengths.

### Iteration steps required to reach system saturation for different population sizes

**Figure 8:**
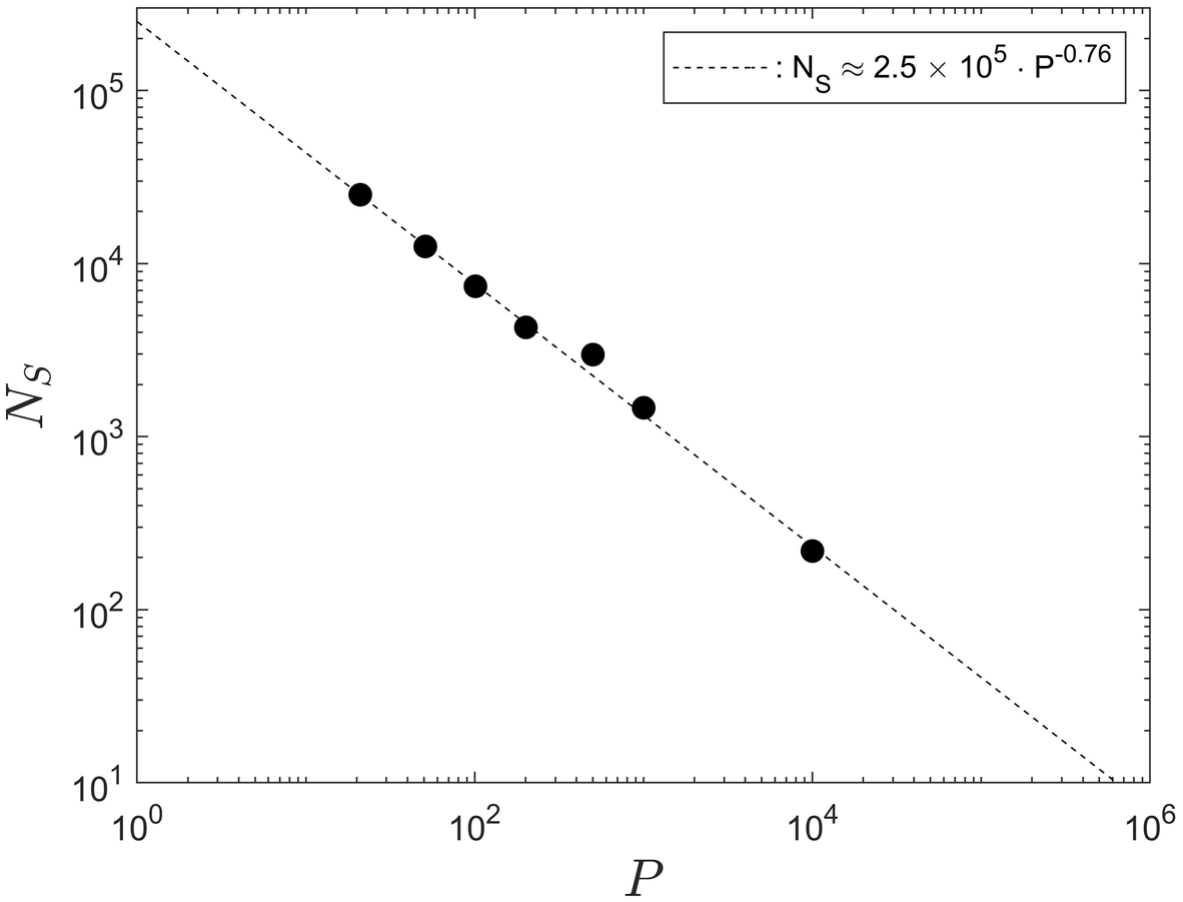
The saturation step, $N_S$ as a function of the population size *P*.

Figure 8 shows the data for the saturation step, $N_S$, from Table 1, as a function of the associated population size, *P*. It can be seen that, in the double-logarithmic representation, the data are very well represented by a straight line over two orders of magnitude in each coordinate direction. This implies the existence of a power law scaling and that, in turn, yields a result of fundamental importance. That is, because power-law scaling implies self-similarity (cf.^19^). Thus, the power-law scaling means that the temporal system development proceeds in the same fundamental manner for any population size *P*.

The line interpolating the data is a least-squares fit given by $N_S \approx 2.5 \times 10^5 \cdot P^{-0.76}$. Provided that the power-law scaling does not break down for populations substantially outside the interval [21, 10001], the data fit implies that for the smallest possible population of $P_1 = 2$ it requires ${\mathcal {O}}(1.48 \times 10^5)$ iteration steps until the single susceptible agent becomes infected by the infection seed. Moreover, for $P_2 \approx 1.3 \times 10^7$ the entire population becomes infected for iteration steps ${\mathcal {O}}(1)$. This would mean that, for the particular contagion radius used in the current simulations, there is a sufficiently large number of agents present such that each one is within the contagion radius of another one at the start of the simulations.

However, the power-law scaling must necessarily break down for very large populations when ony a very small number of iteration steps is required to reach saturation. Similarly, the power law scaling must break down when the population is very small such that a proper function for *I*(*n*) cannot be established. Nevertheless, Fig. 4 and Fig. 8 show, respectively, that the description of the data by means of a hyperbolic-tangent profile and the power-law scaling, of $N_S$ with *P*, remain valid for populations as small as, at least, $P = 21$.

The value of the exponent of $-0. 76$ is very close to $- 3/4$. That makes it tempting to speculate that it might be possible to infer the value based on some scaling arguments. If the problem depended on dimensional quantities, it would be possible, for instance, to conduct a dimensional analysis (cf.^19^). However, there are no proper dimensional quantities involved in the problem.

### Parameters $\alpha$ and $\beta$ for different population sizes

While Fig. 8 displays a convincing power-law scaling for the saturation step, $N_S$, it is not that straightforward to infer a definitive, corresponding scaling for the parameters $\alpha$ and $\beta$. That is, because each one only varies over a very narrow interval ($[2.149 \le \alpha \le 2.512], [0.299 \le \beta \le 0.03382]$), for population sizes in the range [21, 10001] . Therefore, the discussion of the scaling for $\alpha$ and $\beta$, in this section, is somewhat speculative.

**Figure 9:**
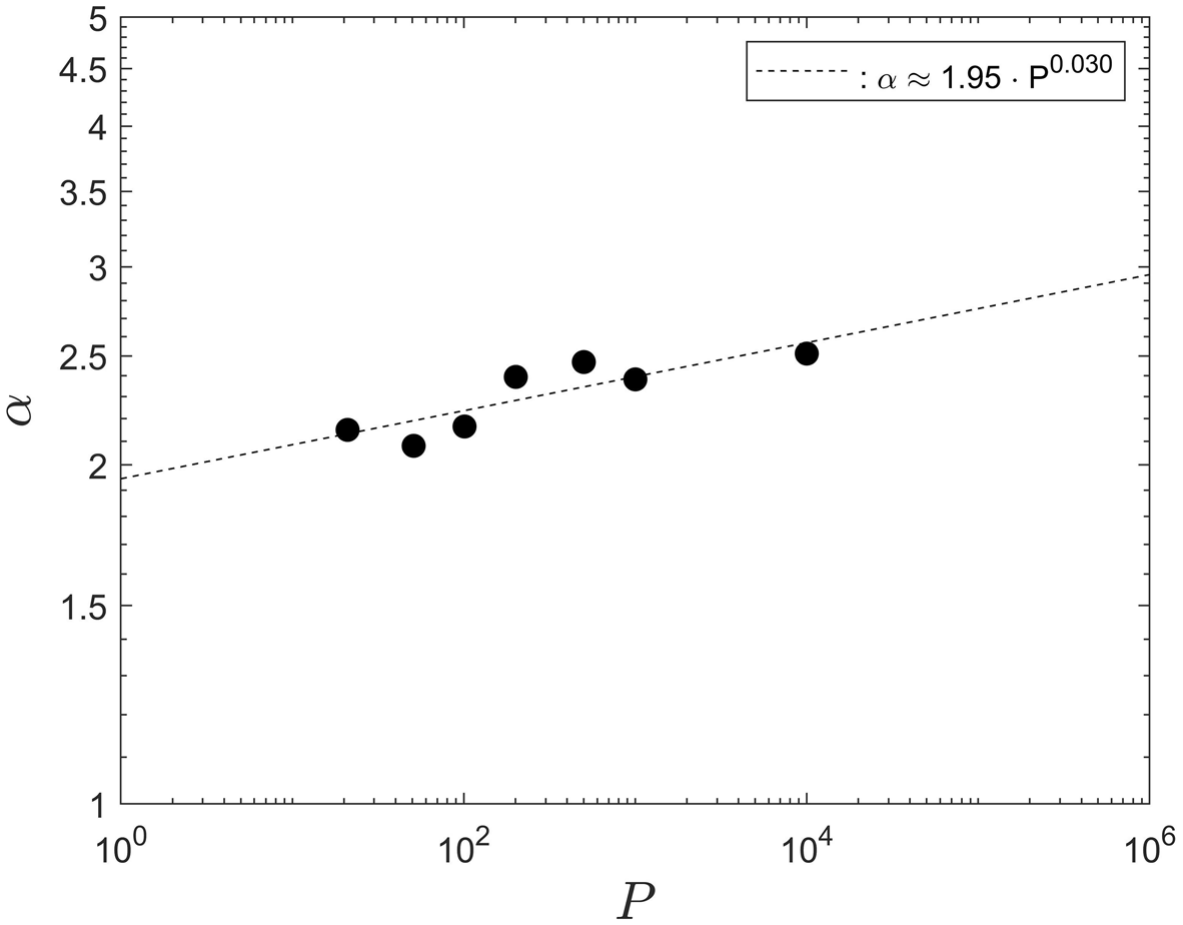
The parameter $\alpha$ as a function of the population size *P*.

**Figure 10:**
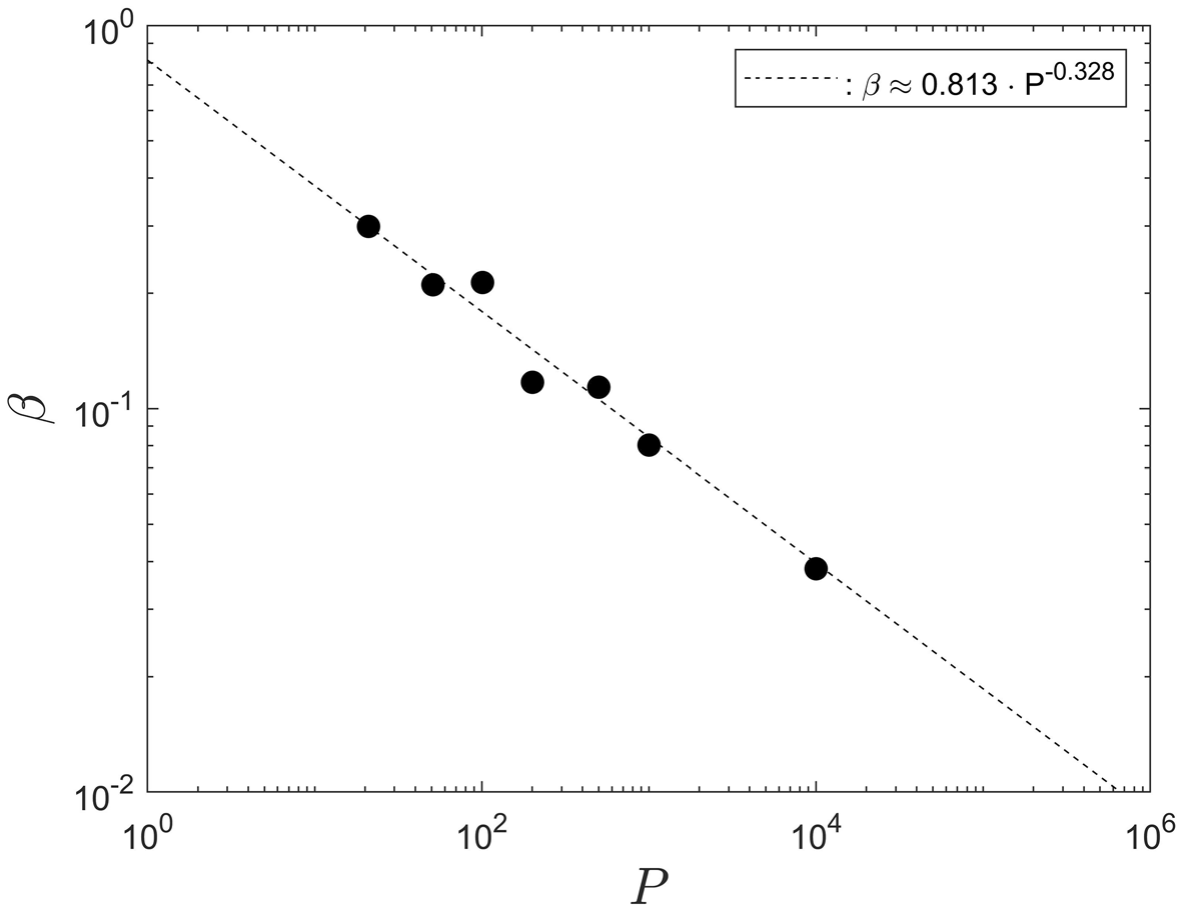
The parameter $\beta$ as a function of the population size *P*.

Since $N_S$ displays a clear power-law scaling, implying self-similarity, it is not unreasonable to expect that power-law scaling also exists for $\alpha$ and $\beta$. Therefore, the data for both parameters, from Table 1, are shown in Figs. 9 and 10 as a function of the associated population size, *P*. It can be seen that, in both cases, the data do not exclude the existence of power-law scaling since they do appear to lie on straight lines, in the double-logarithmic representation.

Assuming that power-law scaling for $\alpha$ and $\beta$ does indeed exist, least-squares data interpolations for the data points in Fig. 9 and 10 yield, respectively, $\alpha \approx 1.95 \cdot P^{0.030}$ and $\beta \approx 0.813 \cdot P^{-0.328}$. For the minimum and maximum populations, $P_1 = 2$ and $P_2 = 1.3 \times 10^7$, would imply $\alpha (P_1) \approx 1.99$ and $\alpha (P_2) \approx 3.19$ and $\beta (P_1) \approx 0.76$ and $\beta (P_2) \approx 3 \times 10^{-3}$.

If the power-law scaling for the fitting parameters $\alpha$ and $\beta$ were to extend substantially beyond the population sizes investigated, it would represent a result of substantial practical relevance. It would mean that it is possible to predict the infection numbers for any population size without conducting any further computational simulations. That is, as long as all other independent system parameters remain unchanged. Nevertheless, even if the power-law scaling for $\alpha$ and $\beta$ does not extend beyond the population sizes investigated, then the least-squares fits still remain to be a useful data interpolation for the interval $21 \le P \le 10001$.

If one, or more, of other system parameters are modified then the data fits from all sections above would have to be adjusted accordingly. Nevertheless, a data analysis analogous to the above can be conducted for each one of the independent parameters, to obtain a complete description of the entire system.

Finally, if the power-law scaling for $\alpha$ were to extend to the largest and smallest populations then one could define a mean value ${\overline{\alpha }}$ to characterize the system as

12 $$\begin{aligned} {\overline{\alpha }} = \frac{1.95}{P_2 - P_1} \int _{P_1}^{P_2} P^{0.03} \,dP \ \ . \end{aligned}$$

For the data considered here this yields ${\overline{\alpha }} \approx 3.19$. The value of ${\overline{\alpha }}$ should represent a characteristic value for the system, for variations of the population size and for the particular values of the other independent input parameters used here. Likewise, the mean value of $\alpha _R \approx 2.31 \pm 0.16$, for the data in Table 1, or the integral of Eq.(12) in the range [21, 10001] , giving ${\overline{\alpha }} \approx 2.57$, is characteristic for that population range. The values of $\alpha _R$ and ${\overline{\alpha }}$ can be used for a quantitative comparison to corresponding values obtained for other models, such as data from Refs.^2–15^. Equivalent mean values can be defined for the fitting parameter $\beta$.

### Iteration steps required to reach system saturation for different infection probabilities and contagion radii

**Figure 11:**
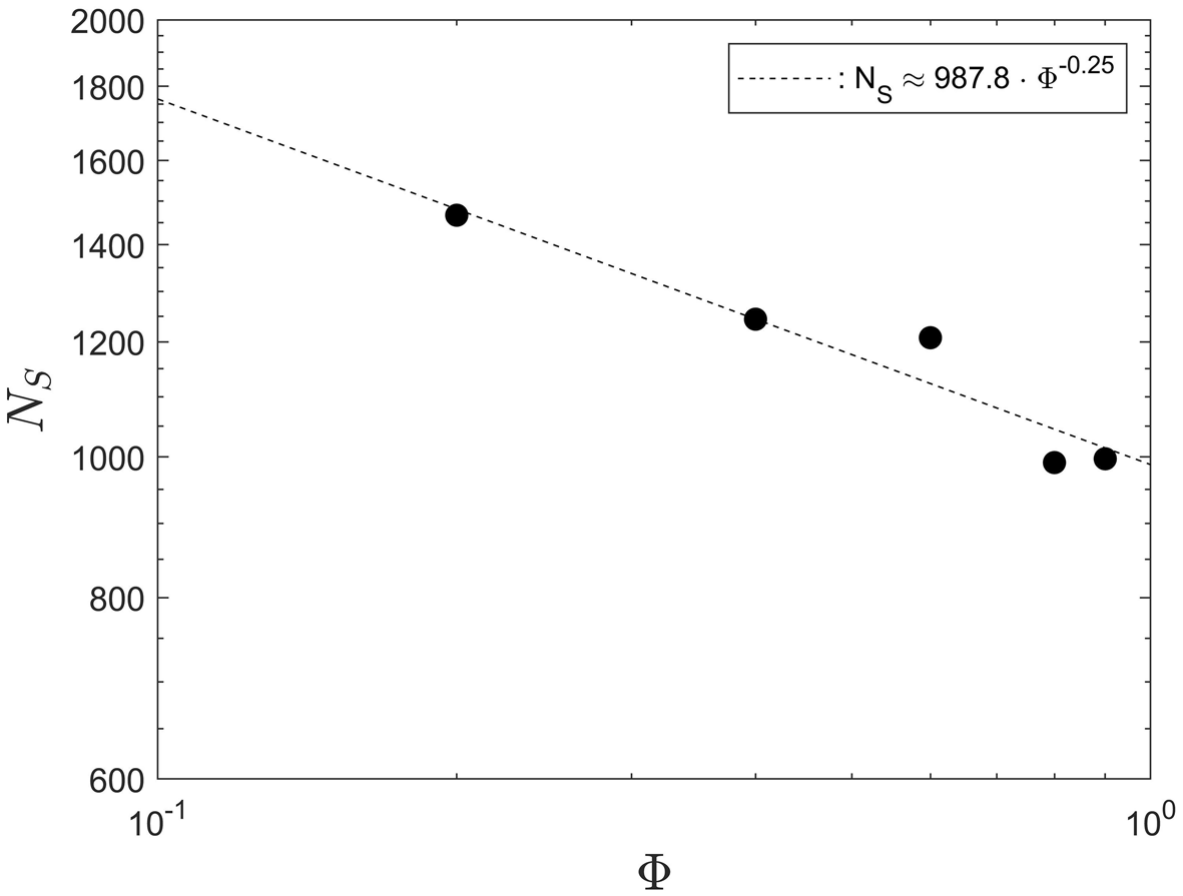
The saturation step, $N_S$ as a function of the infection probability $\Phi$, for a population $P = 1001$.

**Figure 12:**
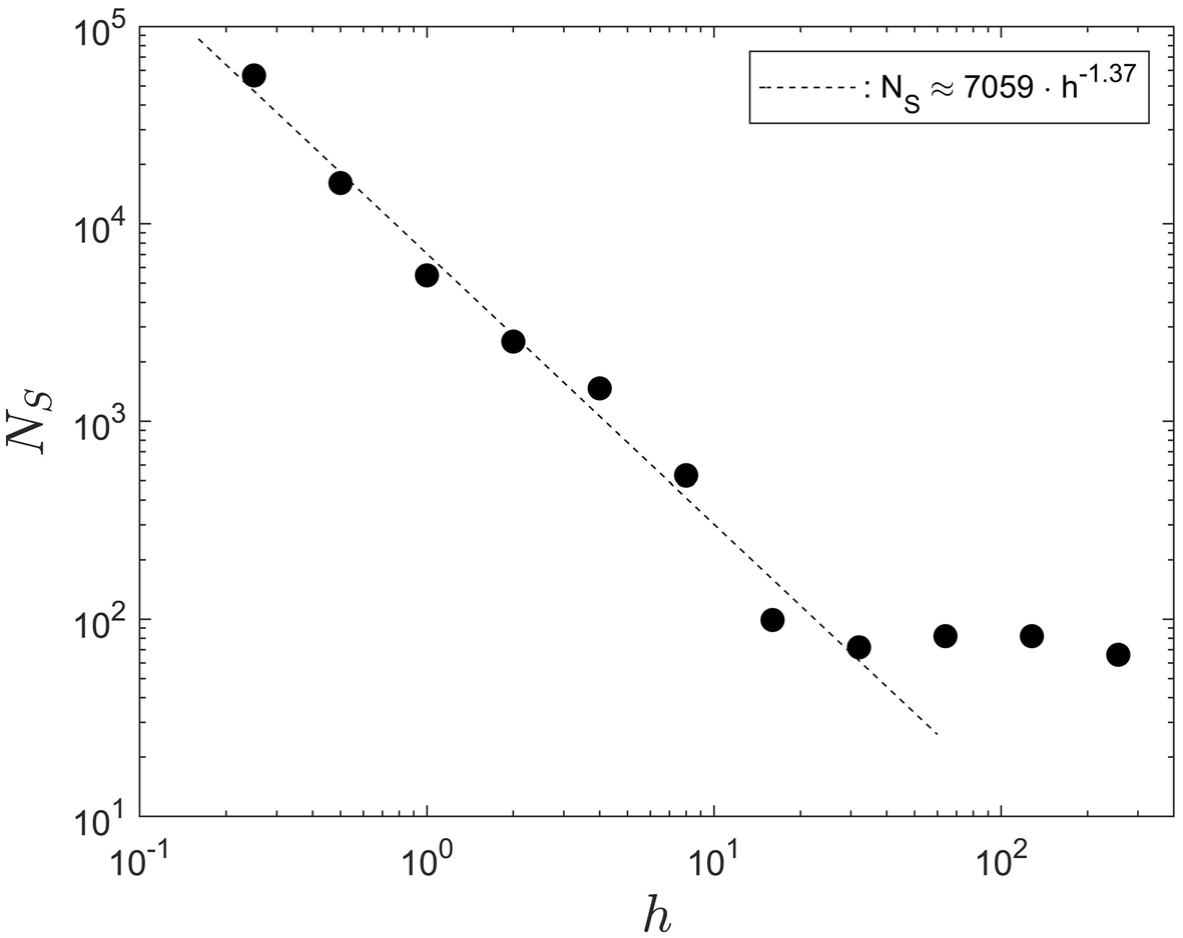
The saturation step, $N_S$ as a function of the contagion radius *h*, for a population $P = 1001$.

Figures 11 and 12 illustrate that the power-law scaling for the saturation step $N_S$ also extends to the variation of the infection probability $\Phi$ and to the contagion radius, *h*. The infection probability $\Phi$, in our notation, is equivalent to the variable *PrInf* in the code in Ref.^16^ (c.f. table in Appendix A). The numeric data associated with Figs. 11 and 12 are summarized in, respectively, Table 2 and 3.

Figure 11 and Table 2 reveal that the variation of the infection probability, in the range $0.2 \le \Phi \le 0.9$, yields saturation steps in the range $1467 \ge N_S \ge 997$. That is a relatively narrow range. Similar to the discussion for $\alpha$ and $\beta$ in “[Sec Sec19]” section it is, therefore, not justified to make a definitive claim that a true power-law scaling exists. Nevertheless, the data points in Fig. 11 lie on a straight line in the double logarithmic data representation and, therefore, they do not contradict the existence of the scaling.

However, the situation is substantially more convincing in Fig. 12, for the data of the variation of $N_S$ with the contagion radius *h* . Here the saturation step varies in the interval $56455 \ge N_S \ge 66$. That is, it varies over three orders of magnitude, with an associated change of the contagion radius *h* of over two orders of magnitude, in the range $0.25 \le h \lesssim 10$.

For the data in Fig. 12 we have varied the contagion radius from very small values, $h = 0.25$ units, to very large values, $h = 256$ units, to explore the entire system dynamics of the code. Values of order of $h = 256$ units are comparable to the size of the computational domain, which is 300 units $\times$ 300 units. Of course, such large values of *h* are not of practical relevance to real-world applications. However, our goal is to explore the overall scaling behaviour of the system and, to that end, such extreme values are considered. In particular note that, by doing so, Fig. 12 clearly reveals the breakdown of the power-law scaling around $h \gtrapprox 10$ when, for further increasing *h*, the value of the saturation step $N_S$ remains essentially constant. That is, the breakdown provides information as regards the range of applicability of the model.

The breakdown of the scaling around $h \approx 10$ can be anticipated. The entire computational domain has a size of 300 units $\times$ 300 units, that is 90, 000 units$^2$. The breakdown of the power-law scaling must be expected when the sum of the contagion regions of the $P = 1001$ agents approaches the size of the computational domain. For the limit radius, $h_l$, when this will be the case that implies 90, 000 units$^2 = P \times \pi {h_l}^2$. Solving the expression for $h_l$ yields $h_l = \sqrt{90,000 / \pi P} \approx 5.3$ units. Therefore, the breakdown of the scaling is expected roughly around ${\mathcal {O}}(h_l=10)$, and that is precisely what Fig. 12 shows.

**Table 2:** Parameters of least-squares fitting, according to Eq. (3), for different infection probabilities $\phi$ and for a population $P = 1001$.

$\Phi$	0.2	0.4	0.6	0.8	0.9
$\alpha$	2.383	2.443	2.454	2.5	2.206
$\beta$	0.0803	0.0921	0.0957	0.1168	0.0417
$N_S$	1467	1244	1208	991	997

**Table 3:** Parameters of least-squares fitting, according to Eq. (3), for different contagion radii *h* and for a population $P = 1001$.

*h*	0.25	0.5	1	2	4	8
$\alpha$	3.876	4.019	3.180	2.642	2.379	2.365
$\beta$	0.2431	0.2209	0.1579	0.0857	0.0809	0.158
$N_S$	56455	16096	5482	2533	1467	533

*h*	16	32	64	128	256	
$\alpha$	3.831	6.571	8.398	7.99	6.232	
$\beta$	0.4059	0.7246	0.844	0.8687	0.8516	
$N_S$	99	72	82	82	66	

A corresponding consideration regarding the area of the population’s combined contagion regions, in comparison to the available computational domain, can also be made for the data in Fig. 8. For the data in that figure the contagion radius was set to $h = 4$ units. Therefore, the computational domain will be filled for a population of roughly $P = 90,000$ units$^2 / \pi (4$ units$)^2 \approx 1406$. Reference to Fig. 8 shows that the power-law scaling persists until the data point $P = 10,001$. The reason for that is, probably, that a computational domain that is tiled by many small contagion regions provides more flexibility for movements of the agents that results in interstitial space than a computational domain that is filled by a small number of areas with a very large contagion radius. Nevertheless, the limit number $P = 1406$ for the data in Fig. 8 implies that for a computational size of 300 units $\times$ 300 units it is not sensible to perform simulations for $P \gtrapprox 10,001$. In that context also note that we found that for the code of Ref.^16^, in its current form, Matlab begins to exceed its array-size limit for populations somewhat larger than $P = 10,001$. Thus, if it were desired to use the code to perform computations for very small contagion radii, but with very large populations it would be required to revise the code structure to overcome that Matlab issue.

Finally, we do not plot the data for $\alpha$ and $\beta$ from Tables 2 and 3. That is, because the data for each of these quantities lie within very narrow intervals and do not reveal any new insight. Note, however, that if one plots these values for $\alpha$ and $\beta$ of Table 3 in a double logarithmic format, then both also display clear evidence of the breakdown of the power-law scaling near ${\mathcal {O}}(h_l=10)$.

## Discussion and conclusion

The scaling behaviour of an agent-based model, originally published in Ref.^1^ to simulate the spreading of infectious diseases, was discussed. The main contribution of the results presented is that the data analysis, as performed here, allows one to draw general conclusions as regards the underlying system behaviour. The analysis presented has yielded simple closed-form expressions to summarize the complex system dynamics. These expressions, in turn, have enabled quantifying how the instant of the maximum infection rate, $N_{Imax}$, of a simulation is related to the knee-point iteration step, $\Lambda$, representing the outbreak end point.

The overriding results, of general nature, of the current summary of results is the scaling behaviour displayed by the data in Figs. 5 and 8,  11, 12. The data in Fig. 5 imply that the fitting parameter $\alpha$ of the hyperbolic tangent profile in Eq. (3) is the quantity relevant to characterize the system development, whereas the parameter $- \beta$ represents the instant of the maximum infection rate. The power-law scaling, of the saturation step $N_S$ with the population size *P*, the infection probability $\Phi$ and the contagion radius *h* in, respectively, Figs. 8, 11 and 12, implies self-similarity, in that the system development proceeds in a similar manner for all population sizes, infection probabilities and contagion radii. That is, the investigated dynamics of the model of Ref.^1^ reproduce themselves in time and space and that represents a very deep property of the system under consideration (cf.^19^).Together with the numeric data for $\alpha$ and $\beta$ in Tables 1, 2 and 3 the entire system dynamics are known for variations of *P*, $\Phi$ and *h*. No further computations have to be performed to predict the behaviour for a variation of any one of these parameters.

Note that the parameters $\alpha$ and $\beta$ are output data, in the sense that they summarize the global effects of the independent input parameters. The agent-based model of^1^ uses similar epidemiological relevant input parameters as, for instance, the methodologically fundamentally different, equation-based approach of^20^ that uses the Richards model^21^. Both models produce growth curves for the number of infected people, as a function of time, to help guide the decision-making processes in connection with monitoring and restricting the spread of a disease.

In^20^, the infection rate, the final epidemic size and time essentially correspond to the infection probability, the population size and the iteration step in the current investigation. The agent-based approach used here provides somewhat more flexibility, in that it also enables independently specifying the contagion radius as an additional input variable. In the case of^20^ one would have to interpret their infection rate as including the combined effects of infection probability and contagion radius. Moreover, the current agent-based approach does not require the shape parameter $\xi$ (cf. Ref.^20^, last sentence of *’Methods’* section, p. 4/17) that is used in^20^ and that, as the authors state, does not have a clear relationship to the epidemiological characteristics.

Nevertheless, the issue to appreciate is that the fitting parameters $\alpha$ and $\beta$ used here summarize the effects of the input parameters after appropriately remapping the simulation data for the number of infected agents, as a function of the iteration step, onto the interval [1, 1] (cf. “Remapping iteration steps” section) and subsequent data interpolations. Through the knowledge of $\alpha$ and $\beta$ the entire dynamics of the model, in its current form, are known and no further simulations need to be performed. The general data analysis methodology applied here will, moreover, allow direct quantitative comparisons of simulation data obtained from different models, and different modelling approaches, for different population sizes and including any other relevant input parameters.

It has been discussed that the hyperbolic-tangent scaling for the number of infected agents must necessarily break down for certain limits of the independent parameters. The scaling has to break down, for instance, for very large population sizes, when the number of iterations steps to saturation is ${\mathcal {O}}(1)$. Similarly, this is the case when the contagion radius becomes so large that the contagion areas of all agents overlap from the start of the simulation. However, the breakdown of the scaling for extreme parameter values is, maybe, of no great practical concern to epidemiology. However, it defines the parameter limits within which the computational code can be applied to yield valid results.

All results of the current study could, in principle, be mapped onto real-world time by means of Eq. (11). The caveat being, as discussed in “Scaling results to real-world time” section, that the real-world time, *T*, until a system reaches saturation is, of course, in practice not known in advance. Nevertheless, a related issue is encountered in connection with the Richards model in Refs.^20,21^ where the time *t* in Eq. (1) of Ref.^20^ is arbitrary and where the abscissa of their Fig. 3 does not specify a unit (e.g. days, weeks, months) for time.

Nevertheless, the data analysis, as presented here, also constitutes a means to quantitatively compare the current results to corresponding data from models by other authors, such as data from Refs.^2–15,20^ and numerous other publications, provided their data were scaled according to those discussed here.

We expect that, if the relevant data of Refs.^2–15^ were analysed according to the methodology adopted here, a general behaviour entirely analogous to that from the current study would be found. The specific numeric values for the relevant parameters required to summarize the system dynamics would be different for all models. However, these numeric values represent a means for a quantitative intercomparison of data from all models.

The data-analysis methodology applied here is not restricted to the increasing number of infected agents at the outbreak of a disease. A corresponding, adapted, methodology can be applied for any scenario of initially increasing and subsequently decreasing numbers of infected agents and other quantities, and scenarios, of interest.

We conclude with a brief comment as regards the origin of the hyperbolic tangent scaling of the data from Ref.^1^. The scaling probably arises as a consequence of the disease spreading representing a process whereby the infection propagates away from the infection seed in terms of a diffusion process. That is, it propagates from a region of higher concentration of infected agents, located in the vicinity of the infection seed, to regions of lower concentration. Such processes are described by reaction-diffusion equations, and these are associated with the propagation of wave fronts^22^. Travelling waves are solutions to nonlinear evolution and wave equations. Most nonlinear wave equations can be treated by the *tanh method* because their solitary wave solutions can be written in terms of the hyperbolic tangent^23,24^.

## Supplementary Information

Supplementary Information.

## Data Availability

The relevant numeric data are included in the article. The code to produce raw data is available at Ref.^16^ cited in the article.

## Code availability

The code used for this study is available online at Ref.^16^ cited in the article. The original code was modified, as described in detail in the article.
