# Supplementary material for: Scaling of agent-based models to evaluate transmission risks of infectious diseases
Source: Sci Rep. 2023 Jan 2;13:75. doi: 10.1038/s41598-022-26552-w (PMC9807651; doi:10.1038/s41598-022-26552-w)
Supplement: Supplementary file 1 — Supplementary Information. [file 41598_2022_26552_MOESM1_ESM.pdf]

Appendix A Parameter values code

Parameter values and ranges, for the code (Ref. [16]) of Ref. [1], that remained constant throughout all simulations discussed herein. The names of the variables in the right column of the table are those used in the code available at Ref. [16].

Number of infected in the popul.	Infected = 1
Distance of contagious	h = 4
Probability of transmission	PrInf = 0.2
Local movement size	S = 2
Size of facility	range = [0 300 0 300]
Probability of contagious	rangeI = [0.1 0.3]
Probability of mobility	rangeM = [0.2 0.4]
